# Green taxes as ecosystem conservation: an analysis of the industrial sector's view in Peru

**DOI:** 10.12688/f1000research.127058.1

**Published:** 2022-11-25

**Authors:** Walter Gregorio Ibarra Fretell, ROSARIO VIOLETA GRIJALVA SALAZAR

**Affiliations:** 1 Universidad Cesar Vallejo, Lima, Peru

**Keywords:** Environmental taxes, ecosystem, environmental problems, pollution, secondary effects.

## Abstract

**Background:** Environmental problems are becoming more recurrent nowadays, which is why many countries have acted on the matter, through different forms, laws and taxes that can contribute and reduce the polluting impact of companies at the time of manufacturing their products. One of the reforms has been environmental taxes, which are not only aimed at raising money, but also at taking corrective action on the behavior of companies that damage both the environment and the health of the population.

The research is intended to finding out the opinion and interest of managers of different companies in the industrial sector on environmental taxes, also known as green taxes, and whether they consider it necessary to add green taxes to the current tax system.

**Method:** For data collection of the research, 120 managers of small and medium-sized enterprises in the industrial sector were questioned about whether green taxes could have an influence on ecosystem conservation and several other questions.

**Results: **63.3% of the managers surveyed agree that the application of an environmental tax is necessary.

**Conclusion:** It is concluded that managers are in favor of green taxes and  approve that  such taxes should be given as part of ecosystem conservation and environmental protection for all businesses, and that they promote businesses that do not generate any side effects or transformations to the environmental balance, thus improving the quality of life of residents.

## Introduction

The greatest threat we are currently facing is climate change. The temperature has increased by 1.1°C in all these years and if this continues, the consequences could be catastrophic. Climate change constitutes a systemic risk that compromises the sustainability of all countries on the planet, however, although its contribution to global pollution is minimal, Peru and the Latin American region are the most vulnerable due to their rich biodiversity and their own endemism ( Rivera, 2022).

The population depends on the existence, in the necessary quantities and quality of the environment, to use them for direct consumption or after conversion through industrial production. However, all these production processes cause pollution from transformation of organic matter to toxic substances ( Bravo *et al*., 2021), which is partly due to the growth of sectors such as industry.

The growth that the industrial sector has had in recent times is imminent and this growth and the improvement in the quality of life of man have caused air, water and soil pollution directed towards, thus causing the depletion of natural resources and their degradation, negatively influencing either directly or indirectly on the welfare of the population ( Evelia *et al*., 2019). That is why we need to reduce pollution, and a tool that can help combat climate change is green or environmental taxes.

Environmental taxes, also called green taxes, have emerged as a useful and necessary tool to combat climate change, and their economic and social implications on tax behaviors that are harmful to the health of the planet in a way that favors sustainable development ( Bedoya *et al*., 2022). Although there is consensus on its potential to curb climate change, the application of this is not so easy and on the contrary, faces resistance, as countries and industries have invested in traditional and polluting technologies.

Countries in Latin America and the Caribbean (LAC) have so far been introducing such taxes. Among these countries, Costa Rica, and Colombia, recognized for their good environmental performance, stand out. The question is, what has Peru done for the introduction of environmental taxes?

In Peru, the government has introduced a tax on the consumption of plastic bags. Although more and more people are becoming aware of the environmental impact, the recycling culture is still far from the daily interest of Peruvian citizens, a fact that is far from the attention of other countries in Europe and even in the Asian continent, and the State has yet to define protocols that benefit the environment ( Dávila & Mena, 2022).

Lima has one of the largest industrial sectors, where companies do not take into account the environmental costs generated by their operations, as the current rules aimed at correcting negative actions are scarce and have little or no effect, as the goods and services produced do not play the role that a socially responsible entity should have in protecting the environment, a vital characteristic today for any company. The research is justified from a socio-economic point of view, because there is a deterioration of the environment in all regions of Peru, caused by industrial activities. Studying the opinion of managers regarding green taxes can have an impact on regulating the activities that generate pollution with a tax that obliges businessmen to take responsibility for the activities they carry out, promoting a sustainable, responsible culture with reasonable use of resources.

In fact, reducing the impact and moving towards sustainable development requires an increasingly significant economic investment, and this action must be linked to the State's environmental policy and legislative action. Through this study we show interest in the need to incorporate green taxes into the Peruvian tax system. The combination of these taxes would provide multiple benefits by reducing existing pollution and making better use of resources, so the opinion of the industrial sector located in Lima on the issue of environmental taxes will be explored.

## Methods

The type of research conducted in this study was applied-descriptive ( Hernández-Sampieri & Mendoza, 2018), quantitative and cross-sectional ( Ñaupas *et al.*, 2018).

According to the report of the Instituto Nacional de Estadística e Informática (2018) there are 1, 106, 853 registered companies in Lima, of which only 5034 (8.5%) are located in South Lima (study scenario). Of this number, 458 (9.2%) are companies belonging to the industrial sector, the latter being the population to be considered for the study.

To determine the sample size, it was calculated based on a probability of success (p) of 0.88, a probability of failure of 0.12, and a confidence level of 95% (Z-value=1.96) and a margin of error of 5% distributed as follows:

$$n=\frac{N*Z^{2}*p*q}{e^{2}*\left(N-1\right)+Z^{2}*p*q}$$

$$n=\frac{458*1.96^{2}*0.88*0.12}{0.05^{2}*\left(458-1\right)+1.96^{2}*0.88*0.12}=120$$

The sample consisted of 120 company managers, which was obtained through simple random sampling where managers and/or owners of companies in the industrial sector were interviewed.

### Data collection technique and instrument

The collection and application of the instruments was carried out from 1 ^st^ October to 15 ^th^ December, 2021, and data processing was carried out from 20 ^th^ December 2021, to 15 ^th^ January 2022. Finally, the presentation and interpretation of results was carried out until 28 ^th^ February 2022.

The data collection technique was the survey and the data collection instrument was a questionnaire.

The questionnaire consists of 14 items divided into three dimensions, with a polytomous response alternative, which aims to identify the opinion of the study sample on environmental taxes for the industrial sector.

### Dimensions

• Economic and social aspect - items 1, 2, 3, 4 and 5
• Environmental policy - items 6, 7 and 8
• Environmental impact - items 9, 10 and 11
• Sustainable development - items 12, 13 and 14

The response alternatives were: (5) Strongly agree, (4) agree, (3) neither agree nor disagree, (2) disagree, (1) strongly disagree.

### Data processing methods

The researchers went directly to the management of each company and a questionnaire was administered to each manager. The managers were the ones who filled out the questionnaire freely and without the intervention of the interviewers. After the instrument had been applied to the 120 managers in the sample, all the information was passed directly to the SPSS version 25.0 statistical program (RRID:SCR_002865), all the descriptive data processing and analysis of the data obtained was carried out, and all the information was presented in the form of a frequency table, which provided a response to the objective of the study.

It should be noted that none of the managers surveyed refused to participate in the research, nor did they drop out of the questionnaire during or after the application of the questionnaire, so no data is missing from the research.

### Reliability

The Cronbach's Alpha test was used to measure the reliability of the instrument (α>0.7), obtaining a value of 0.791, concluding that the reliability of the instrument is high.

### Ethical approval

The study was approved in July 2021 by the Vice-Rector of Research of the Universidad César Vallejo, through registration No.190-2021-VI-UCV.

### Consent

Written informed consent was obtained from all participants. Each manager and/or owner received a consent form disclosing all information related to the development of the questionnaire, which they were required to read and sign and then return to the researchers.

Those who agreed to participate and signed the informed consent form were informed that their personal data would remain anonymous according to Law N° 29733-Law on the protection of personal data ( Diario el Peruano, 2011).

## Results

Table 1 shows that the industrial sector shows a tendency to be in favor of changes to help the environment; 63.3% of the managers surveyed agreed that the application of an environmental tax is necessary and that companies in the industrial sector have the economic capacity to pay an environmental tax (67.5%).

**Table 1.** Industrial companies' views on green taxes if they are added to the tax system.

Questions	Responses	Frequency	Percentage
In your opinion, is a tax necessary to protect the environment?	Strongly disagree	0	0.0%
	Disagree	0	0.0%
	Neither disagree nor agree	5	4.2%
	Agree	39	32.5%
	Strongly agree	76	63.3%
Do you think it is necessary for tax pollution generated by companies in the industrial sector?	Strongly disagree	0	0.0%
	Disagree	0	0.0%
	Neither disagree nor agree	0	0.0%
	Agree	38	31.7%
	Strongly agree	82	68.3%
Do companies in the industrial sector have the economic capacity to contribute to the environment?	Strongly disagree	0	0.0%
	Disagree	0	0.0%
	Neither disagree nor agree	6	5.0%
	Agree	33	27.5%
	Strongly agree	81	67.5%
Do you consider that companies in the industrial sector have the capacity to pay an environmental tax?	Strongly disagree	0	0.0%
	Disagree	0	0.0%
	Neither disagree nor agree	0	0.0%
	Agree	38	31.7%
	Strongly agree	82	68.3%
Do you think that companies in the industrial sector can respect the maximum permissible pollution limits?	Strongly disagree	0	0.0%
	Disagree	0	0.0%
	Neither disagree nor agree	20	16.7%
	Agree	58	48.3%
	Strongly agree	42	35.0%
Do you think that placing an environmental tax in the Peruvian tax system would reduce the degree of pollution generated by industrial companies?	Strongly disagree	0	0.0%
	Disagree	42	35.0%
	Neither disagree nor agree	17	14.2%
	Agree	37	30.8%
	Strongly agree	24	20.0%
Will companies in the industrial sector internalize the environmental costs they generate within their production processes?	Strongly disagree	6	5.0%
	Disagree	13	10.8%
	Neither disagree nor agree	25	20.8%
	Agree	32	26.7%
	Strongly agree	44	36.7%
When establishing the tax obligation, do you think that companies would improve controls within the production processes with respect to the use of pollutants?	Strongly disagree	0	0.0%
	Disagree	6	5.0%
	Neither disagree nor agree	16	13.3%
	Agree	67	55.8%
	Strongly agree	31	25.8%
Do you believe that the application of an environmental tax liability would lead to a positive change in production patterns in industrial sector companies to protect the environment?	Strongly disagree	0	0.0%
	Disagree	0	0.0%
	Neither disagree nor agree	27	22.5%
	Agree	81	67.5%
	Strongly agree	12	10.0%
Do you think that the application of an environmental tax obligation will influence the consumer's choice of a product, knowing that the company complies with environmental protection standards?	Strongly disagree	0	0.0%
	Disagree	0	0.0%
	Neither disagree nor agree	32	26.7%
	Agree	46	38.6%
	Strongly agree	42	35.0%
Do you think that companies in the industrial sector would optimize the technology used or acquire new technology to become more efficient and competitive in the market?	Strongly disagree	0	0.0%
	Disagree	3	2.5%
	Neither disagree nor agree	7	5.8%
	Agree	86	71.7%
	Strongly agree	24	20.0%
Do you think that companies in the industrial sector would be willing to invest in cleaner technologies for their production processes rather than pay environmental taxes?	Strongly disagree	0	0.0%
	Disagree	9	7.5%
	Neither disagree nor agree	12	10.0%
	Agree	75	62.5%
	Strongly agree	24	20.0%
Do you think that establishing an environmental tax would allow for more sustainable development at the national level?	Strongly disagree	0	0.0%
	Disagree	15	12.5%
	Neither disagree nor agree	19	15.8%
	Agree	61	50.8%
	Strongly agree	25	20.8%
Do you believe that pollution reduction and environmental protection can be achieved through the establishment of an environmental tax in the Peruvian tax system?	Strongly disagree	0	0.0%
	Disagree	10	8.3%
	Neither disagree nor agree	25	20.8%
	Agree	34	28.3%
	Strongly agree	51	42.5%

They also considered that it could be difficult for companies to internalize the environmental costs they generate in their production processes (36.6%) and indicated that the application of this tax would not reduce the degree of pollution of industrial companies (35.0%). The addition of a tax is not enough to solve the environmental problem but making this modification would help minimize the impacts. However, a large group considered that companies would prefer to invest in cleaner technology within their processes (62.5%), rather than paying a tax. For all the options, respondents considered that implementing them would allow for sustainable development within the country (50.8%).

## Discussion and conclusion

The work did not present any major constraints that could affect its full development.

It is evident that the majority of the managers agreed (97.5%) with the implementation of an environmental tax, considering that the application and levying of a green tax could help the conservation of the environment, however, the tax alone would not reduce the pollution of the environment today. This must go hand in hand with state policies and collection by the government, all managed by a competent body that controls the systems of funds to be implemented. The idea is that green taxes should be a taxation tool that generates protection, production and conservation systems within the region, and as per Bedoya *et al*. (2022), “green taxation tools should be accompanied by fiscal incentives and facilitation of access to cleaner technologies, energy transition plans for industries and electricity generation and targeted social sustainability to mitigate the social impacts that these can cause” and that the placement of a tax like this should be taken as an opportunity to mobilize efforts and guidelines for the correct direction of the funds that are collected in all tax systems that a country has.

It is understood that the respondents considered that companies that have the economic capacity to pay an environmental tax, the payment of a levy would generate a greater awareness of pollution and that they could identify within their production processes those that generate more pollution, to the point that they internalize their environmental costs and expenditure on new, less pollution-causing technology. The aim is to be a less polluting and ecologically aware organization, all of which would benefit the surrounding agents (such as internal and external clients, competitors, local government and the community in general) by reducing the polluting agents that may arise from the economic activities they carry out.

There is still a long way to go for the government, as so far, it has not identified or designed any type of environmental tax within its public policies, and even more so when these green taxation tools must be accompanied by fiscal incentives and facilitation of access to cleaner technologies, energy transition plans for industries and electricity generation, and targeted social support to mitigate the social impacts that these can cause. This is an opportunity to mobilize efforts and identify from how the collection will be done to where the funds raised through these schemes should be directed.

The conclusion is that the managers of small and medium-sized enterprises in the industrial sector are positive about the introduction of green taxes within the Peruvian framework, which could help conserve the environment. However, this will not be achieved by simply collecting taxes, which will not improve the ecosystem, but must be accompanied by government policies that help to channel all these funds into environmental development and conservation works, generating a more balanced quality of life for the community and wildlife.

## Data Availability

Zenodo. Green taxes as ecosystem conservation: an analysis of the industrial sector's view in Peru. https://doi.org/10.5281/zenodo.7302007 Vr2 ( Ibarra & Grijalva, 2022). This project contains the following underlying data:
• Green taxes database.csv • Green taxes database.sav Zenodo. Green taxes as ecosystem conservation: an analysis of the industrial sector's view in Peru. https://doi.org/10.5281/zenodo.7302007 Vr2 ( Ibarra & Grijalva, 2022). This project contains the following extended data:
• Green taxes for environmental conservation questionnaire.docx • STROBE-checklist-v4-cross-sectional.docx Data are available under the terms of the Creative Commons Zero “No rights reserved” data waiver (CC0 1.0 Public domain dedication).

## References

[ref1] Bedoya C Miranda P Moreno L : *Tributación ambiental, financiamiento climático y flujos ilícitos en América Latina.* 2022. Reference Source

[ref2] Bravo O Osorio M Loor X : *La calidad del desarrollo industrial y su impacto en el medio ambiente.* 2021. Reference Source

[ref3] Dávila J Mena J : Los impuestos verdes y su relación con el derecho fundamental a un medio ambiente saludable. *TecnoHumanismo. Revista Científica.* 2022;2(3):35–66. Reference Source

[ref4] El Peruano D : *Ley de protección de datos personales – Ley N° 29733.* 2011. Reference Source

[ref5] Evelia L Bernal P Herrera A : *Sustentabilidad y gestión Ambiental.* 2019. Reference Source

[ref6] Hernández-Sampieri R Mendoza C : *Metodología de la investigación. Las rutas cuantitativa, cualitativa y mixta.* México: Grupo editorial Mc Graw Hill Education;2018.

[ref7] Ibarra W Grijalva R : Green taxes as ecosystem conservation: an analysis of the industrial sector's view in Peru. Vr.2.[Dataset]. *Zenodo.* 2022. 10.5281/zenodo.7302007 PMC10375052 37521515

[ref8] Instituto Nacional de Estadística e Informática: *Análisis de la Estructura Empresarial de Lima Metropolitana V.* 2018. [Archive PDF]. Reference Source

[ref9] Ñaupas H Valdivia M Palacios J : *Metodología de la investigación cuantitativa – cualitativa y redacción de tesis.* 5ta ed. Colombia: Ediciones de la U;2018.

[ref10] Rivera L : *El cambio climático y la tributación ambiental en el Perú: la reforma pendiente.* 2022. Reference Source

