# Supplementary material for: Green taxes as ecosystem conservation: an analysis of the industrial sector's view in Peru
Source: F1000Res. 2022 Nov 25;11:1381. [Version 1] doi: 10.12688/f1000research.127058.1 (PMC10375052; doi:10.12688/f1000research.127058.1)
Supplement: Peer review history

# Peer review: Reviewer response for version 1

**DOI:** 10.5256/f1000research.139526.r184512

**Published:** 2023-07-27

**Authors:** Jarmo Vehmas

**Affiliations:** 1 Finland Futures Research Centre, University of Turku, Åkerlundinkatu, Finland

The article is a nice generic opinion poll on environmental taxation. There are a few essential points to improve the paper.

1. The focus and design of a specific tax are much more important in the introduction and implementation process because then the real impacts of a proposed tax can be assessed. This perspective deserves to be mentioned in the article.
2. The list of references includes only publications in Spanish. The large literature available in English has not been used. In Western countries, environmental taxation has not been a major political issue recently. Therefore most of the scientific literature is rather old. However, important topics have been the ex-ante impacts of specific environmental tax instrument designs, and ex-post evaluations of the implemented environmental taxes, such as carbon/CO2 and energy tax systems.
3. The international context and differences between the Western countries and other parts of the World such as Latin America and Peru in particular could be brought out. 
4. All background information of the respondent companies is missing in the article, and background questions cannot be found in the attached questionnaire data. This is the most serious problem in the article because differences in answers between various industrial branches cannot be assessed.

Is the work clearly and accurately presented and does it cite the current literature?

Partly

If applicable, is the statistical analysis and its interpretation appropriate?

Not applicable

Are all the source data underlying the results available to ensure full reproducibility?

Yes

Is the study design appropriate and is the work technically sound?

Partly

Are the conclusions drawn adequately supported by the results?

Yes

Are sufficient details of methods and analysis provided to allow replication by others?

Yes

Reviewer Expertise:

Sustainable development, futures studies, environmental and energy policies, sustainability evaluation, decomposition analysis

I confirm that I have read this submission and believe that I have an appropriate level of expertise to confirm that it is of an acceptable scientific standard, however I have significant reservations, as outlined above.

# Peer review: Reviewer response for version 1

**DOI:** 10.5256/f1000research.139526.r184513

**Published:** 2023-07-26

**Authors:** Shamal Chandra Karmaker

**Affiliations:** 1 University of Dhaka, Dhaka, Dhaka Division, Bangladesh

The manuscript entitled "Green taxes as ecosystem conservation: an analysis of the industrial sector's view in Peru" has been investigated in detail. The topic addressed in the manuscript is exciting, however, the authors should address the following issues:

 1. The authors should address the research gap and novelty of the study clearly.

 2. In this study, the sample (manager) was selected using simple random sampling. Is a simple random approach appropriate to ensure representativeness? Why not a stratified random sampling approach?

 3. The Introduction section needs to revise providing a more accurate and informative recent literature review. The authors should cite the following recent articles:

- Karmaker *et al. *(2021 ^1^).
- Singha *et al.* (2022 ^2^).
- Singha *et al.* (2023 ^3^).

5. The manuscript looks like a survey report. The authors should use extensive statistical analysis (Ordered logistic regression or other logistic regression) to ensure the study results.

 6.  "Discussion" section should be added in a more highlighting, argumentative way. The authors should analyze the reason why the tested results are achieved.

Is the work clearly and accurately presented and does it cite the current literature?

Partly

If applicable, is the statistical analysis and its interpretation appropriate?

No

Are all the source data underlying the results available to ensure full reproducibility?

Yes

Is the study design appropriate and is the work technically sound?

Partly

Are the conclusions drawn adequately supported by the results?

Yes

Are sufficient details of methods and analysis provided to allow replication by others?

Yes

Reviewer Expertise:

Statistical modeling, energy economics, econometrics, climate change

I confirm that I have read this submission and believe that I have an appropriate level of expertise to confirm that it is of an acceptable scientific standard, however I have significant reservations, as outlined above.

## References

[1] : The role of environmental taxes on technological innovation. *Energy* .2021;232: 10.1016/j.energy.2021.121052 10.1016/j.energy.2021.121052

[2] : Water conservation behavior: Exploring the role of social, psychological, and behavioral determinants. *J Environ Manage* .2022;317: 10.1016/j.jenvman.2022.115484 115484 10.1016/j.jenvman.2022.115484 35751281

[3] : Quantifying the direct and indirect effect of socio-psychological and behavioral factors on residential water conservation behavior and consumption in Japan. *Resources, Conservation and Recycling* .2023;190: 10.1016/j.resconrec.2022.106816 10.1016/j.resconrec.2022.106816

# Peer review: Reviewer response for version 1

**DOI:** 10.5256/f1000research.139526.r170207

**Published:** 2023-05-04

**Authors:** Alex Borodin

**Affiliations:** 1 Department of Sustainable Development Finance, Plekhanov Russian University of Economics, Moscow, Russian Federation

The study was carried out on a topical issue. The issue of applying green taxes is relevant for both developing and developed countries. The article is a sociological study of the profile group of the population of Peru - entrepreneurs. It is gratifying that most of them are ready for "green" changes. However, it is noteworthy that, along with the readiness for change, there is also an impossibility, bias in the forecast costs for the "green" transformation.

 Perhaps a larger visualization of the data in graphical form would make it possible to more clearly present the results of the study.

 In general, the article is a complete study with an appropriate style and composition. Therefore, it is recommended for indexing.

Is the work clearly and accurately presented and does it cite the current literature?

Yes

If applicable, is the statistical analysis and its interpretation appropriate?

Yes

Are all the source data underlying the results available to ensure full reproducibility?

Yes

Is the study design appropriate and is the work technically sound?

Yes

Are the conclusions drawn adequately supported by the results?

Yes

Are sufficient details of methods and analysis provided to allow replication by others?

Yes

Reviewer Expertise:

social science

I confirm that I have read this submission and believe that I have an appropriate level of expertise to confirm that it is of an acceptable scientific standard.
